# Salivary Melatonin Change in Oral Lichen Planus Patients: A Pilot Study

**DOI:** 10.7759/cureus.95039

**Published:** 2025-10-21

**Authors:** Bina Kashyap, Gaganjot Kaur, Sridhar Reddy Padala, Smita S Birajdar, Rajiv S Desai

**Affiliations:** 1 Oral Pathology, Agartala Government Dental College, Agartala, IND; 2 Oral Pathology, Shaheed Kartar Singh Sarabha Dental College and Hospital, Ludhiana, IND; 3 Oral and Maxillofacial Surgery, Agartala Government Dental College, Agartala, IND; 4 Oral and Maxillofacial Pathology, Vishnu Dental College, Bhimavaram, IND; 5 Oral and Maxillofacial Pathology, Nair Hospital Dental College, Mumbai, IND

**Keywords:** inflammation, melatonin, oral disease, oral lichen planus., oral mucosa, saliva

## Abstract

Objective: Melatonin, a chronobiotic hormone, exerts a variety of biological functions. Very few studies of melatonin in the human oral mucosal tissues and oral diseases under chronic inflammation have been reported. This study aimed to investigate and assess the salivary melatonin levels in oral lichen planus (OLP), a chronic inflammatory disease affecting the oral mucosal surfaces and presenting with varied oral symptoms and clinical presentations.

Methods: The study included 60 patients divided into four groups: healthy controls (15), periodontitis (15), keratotic oral lichen planus (K-OLP; 15), and non-keratotic oral lichen planus (NK-OLP; 15). Pocket depth and interdental clinical attachment loss were measured in all the patients. Unstimulated saliva samples were collected, centrifuged, and analyzed for melatonin using an enzyme-linked immunosorbent assay (ELISA) kit for all patients.

Results: Salivary flow rate and salivary melatonin level consistently decreased from healthy controls to periodontitis, K-OLP, and NK-OLP. The salivary melatonin difference was more between controls and NK-OLP (p<0.05). Periodontitis patients showed more difference in pocket depth and interdental clinical attachment loss than other groups.

Conclusions: Decreased salivary flow and melatonin levels in OLP patients imply that salivary melatonin cytoprotective action in chronic inflammation-generated oxidative stress is interrupted. Hence, such alteration can affect the oral mucosa of OLP patients and contribute to various clinical and oral symptoms.

## Introduction

Oral lichen planus (OLP) is a chronic inflammatory disease with premalignant potential affecting oral mucosal surfaces. Clinical manifestations of OLP consist of asymptomatic keratotic forms (reticular, papular, plaque-like lesions) and symptomatic non-keratotic forms (atrophic, erosive (ulceration), and bullous-like lesions). The exact etiology of OLP is unclear, although immune dysfunction is considered one of the causes of the development of OLP [1]. OLP has been taken into consideration due to its association with an increased chance of oral cancer [2]. However, the underlying mechanisms of OLP that initiate the development of oral cancer have not been established. Existing evidence suggests that the chronic inflammatory process in OLP can influence cell proliferation, differentiation, and survival, and contributes to cancer initiation, progression, invasion, and metastasis [3].

Melatonin is an endogenous hormone, synthesized by the pineal gland, and its main function is the regulation of the circadian rhythm (day-night cycles). Pinealocytes are the main cells responsible for producing and secreting melatonin via neural pathway, when the suprachiasmatic nucleus, in the hypothalamus stimulates the pineal gland [4]. Melatonin exerts a variety of biological functions. It plays a cytoprotective role under physiological conditions in the oral mucosa via various molecular mechanisms, including directly scavenging radicals, binding to membrane receptors, and interacting with cytosolic and nuclear proteins [5]. In pathological conditions, melatonin can enhance pro-oxidant, and pro-apoptotic actions to cause cell damage and malignancy. Melatonin functions as a conditional pro-oxidant/pro-apoptotic through the generation of reactive oxygen species (ROS) in several tumoral or non-tumoral cells. It promotes ROS production either by interacting with calmodulin or the mitochondrial pore/complex. However, the pro-oxidant action of melatonin needs to be established [5]. It is suggested that melatonin, which is not bound to albumin, diffuses passively into the saliva, and enters the oral cavity as free melatonin [6]. Earlier, the origin and source of salivary melatonin was thought to be from blood, but recent research showed melatonin synthesis from the salivary gland. Hence, it was clear that the salivary gland synthesizes melatonin released in saliva [6,7]. In the oral cavity, melatonin is an antioxidant and anti-inflammatory agent, rather than a hormone [8]. Melatonin is an immunomodulator, the synthesis inhibition of which causes a suppressed humoral and cellular response. It has also shown a strong anti-cancer effect [9]. Melatonin can be detected in the blood, but its presence in saliva, gingival tissue, and gingival crevicular fluid (GCF) was controversial earlier. Melatonin after being synthesized from the pineal gland, reaches the systemic circulation and hence can be detected in blood and plasma [10]. The presence of melatonin in gingival tissue and GCF is considered an extra pineal site for melatonin biosynthesis [11].

The importance of melatonin has not been established completely in the oral cavity. Few studies have shown its protective role in periodontal disease. The inflammatory component in periodontitis has an impact on oxidative and antioxidant levels, that in turn affects salivary melatonin levels [12,13]. Melatonin can help regress the symptoms of viral and fungal infections and prevent oral ulcers. The presence of melatonin in the oral mucosal epithelium helps in oral epithelial homeostasis [14]. Determination of salivary melatonin is a non-invasive approach, and it has great diagnostic potential in reflecting the secretory profile of the melatonin rhythm. Considering salivary melatonin effects on the periodontium, we assumed that melatonin alteration is also induced in OLP. The alteration reflected in the salivary melatonin profile in OLP can be an early diagnostic marker of OLP and several other oral diseases. Hence, our study aims to assess the salivary melatonin level in OLP patients and healthy controls. We hypothesize that chronic inflammation in OLP can alter salivary melatonin levels and can provide new information regarding the pathogenesis of OLP progressions.

## Materials and methods

Study design

**Figure 1:**
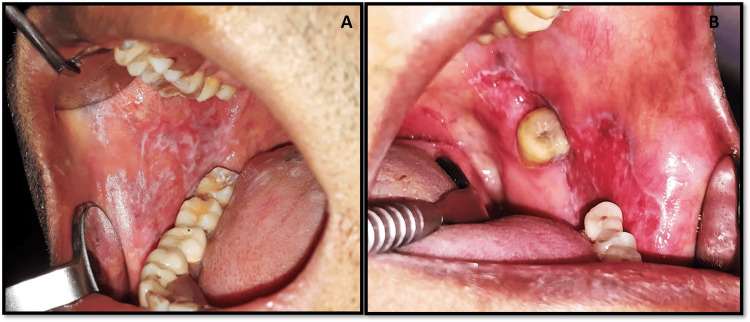
Clinical presentation of oral lichen planus (OLP) – (A) keratotic oral lichen planus (reticular) and (B) non-keratotic oral lichen planus (erythematous) Image credit: R. S Desai

The study was conducted between the period from January 2022 to May 2023. A total of 60 patients between the ages of 21 and 75 years were included in the study. All patients with a history of any other medical diseases and smoking habits were excluded from this study. The patients were distributed further into four groups; healthy controls (15), patients with periodontitis (15), keratotic oral lichen planus (K-OLP; 15), and non-keratotic oral lichen planus (NK-OLP; 15). Periodontitis groups (15) were defined as interdental clinical attachment loss (CAL) equal to or greater than two in nonadjacent teeth OR buccal/oral CAL ≥ 3 mm with pocketing > 3 mm detected at ≥ 2 teeth [15]. The 30 OLP patients were categorized by the American Academy of Oral and Maxillofacial Pathology (AAOMP) criteria [16] into 15 keratotic (reticular) and 15 non-keratotic (atrophic/erythematous) OLPs (Figure 1). All the patients were informed about the study and the enrolled participants were asked to sign an informed consent before the start of the study. The Ethical Committee of the Institute designed and approved the observational case-control study with reference number SKSS-DC-2K25/856 - 278. The study was conducted following the Declaration of Helsinki. 

Sample collection and melatonin assay

The procedure for saliva collection was explained to each participant. Salivary samples were collected in the early morning between 8 and 9:30 am because the melatonin levels are at peak at night and reduce during the day. Whole unstimulated saliva was collected, for which the participants were asked to refrain from eating and drinking for two hours before sampling. To ensure the removal of any possible debris or contaminating materials in the oral cavity, the participants were also asked to thoroughly wash his/her mouth with water and wait a few minutes for clearance. Plastic vials were provided to the subjects to collect saliva at rest. They were asked to relax and bend their head down to collect saliva into a sterile vial. The salivary flow rate was calculated and was represented in mL/min. To prevent bacterial growth, the samples were stored in a compact cooling box. Then, the samples were centrifuged at 4000 rpm for 15 minutes at 4 degrees Celsius, and supernatants were collected and stored at -70°C until analyzed by enzyme-linked immunosorbent assay (ELISA).

Melatonin concentration in the saliva was detected through an ELISA kit (MyBioSource Systems, CA, San Diego, USA). The sandwich ELISA technique was performed according to the manufacturer's instructions. The ELISA kit provided an assay sensitivity of < 1.56 picograms per milliliter (Pg/ml). The total salivary melatonin concentration level was obtained with a spectrophotometer at 450±10 nm wavelength.

Statistical analysis

The results obtained were statistically analyzed using IBM SPSS statistic version 26 for Windows (IBM Corp., Armonk, NY, USA). The standard deviation (SD) and mean were used for nominal variables. The Shapiro-Wilk test was applied to assess the normality of the parameters. Independent t-test and one-way analysis of variance (ANOVA) with the least significant difference test was used further. P-value < 0.05 is considered statistically significant.

## Results

Clinical findings

**Figure 2:**
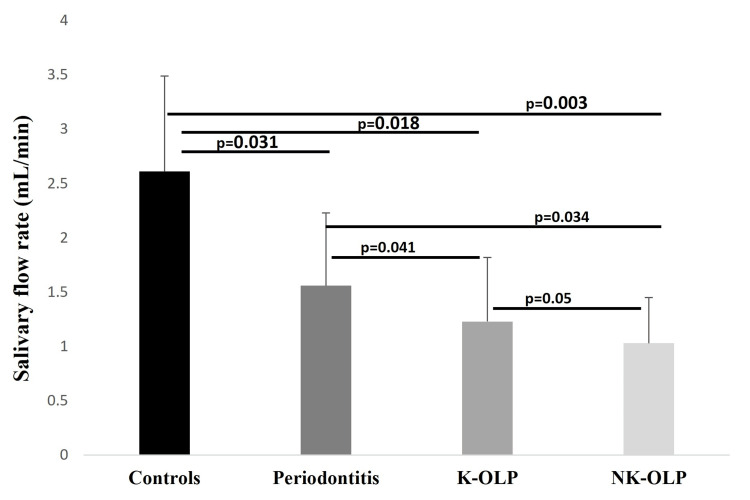
The salivary flow rate differences between controls, periodontitis, keratotic oral lichen planus (K-OLP), and non-keratotic oral lichen planus (NK- OLP).

The age range of the healthy controls, periodontitis, and both OLP groups are presented in Table 1. There were more females (N=38) than males (N=22) in the study sample. There was a non-significant difference in gender among study groups (P-value = 0.091). The salivary flow comparison among study groups showed a gradual decrease from controls to periodontitis to K-OLP and NK-OLP (p=0.02). Controls (mean ± SD; 2.61 ± 0.88) showed a very high difference in salivary flow rate compared to NK-OLP (mean ± SD; 1.03 ± 0.42) than other groups. The difference in the salivary flow was also greater between periodontitis (mean ± SD; 1.56 ± 0.67) and K-OLP (mean ± SD; 1.23 ± 0.59); periodontitis and NK-OLP; and K-OLP and NK-OLP (Figure 2). The age of the patient and the salivary flow did not show any association (p=0.084). The present study's findings showed a higher mean value of periodontal pocket depth (PPD) measurements in the periodontitis group compared to other groups (p=0.034). Although, no significant differences between other groups were observed. Similarly, the periodontitis patient group presented with a higher mean value of CAL measurements than the other groups (p=0.041). However, the notable differences observed between controls, K-OLP, and NK-OLP groups were not statistically significant.

**Table 1:** Demographic data and clinical findings of the study groups. N – Number of subjects; M – Male; F – Female; CAL – Clinical attachment loss; K-OLP – Keratotic oral lichen planus; NK-OLP – Non-keratotic oral lichen planus; *p<0.05 between periodontitis and other groups (controls, K-OLP and NK-OLP).

Study groups	Age in years (mean ± SD)	Pocket depth (mean ± SD)	CAL (mean ± SD)
Healthy controls (N = 15; 6M/9F)	22–70yrs (39 ± 3.9)	2.09 ± 0.08	1.19 ± 0.17
Periodontitis (N = 15; 7M/8F)	24 – 69yrs (42 ± 4.6)	3.80 ± 0.63*	2.62 ± 0.71*
K - OLP (N = 15; 5M/10F)	23–71yrs (41 ± 3.7)	2.42 ± 0.23	1.42 ± 0.21
NK - OLP (N = 15; 4M/11F)	23 – 71yrs (41 ± 3.7)	2.71 ± 0.33	1.45 ± 0.14

Salivary melatonin levels in controls, periodontitis, and OLP groups

**Figure 3:**
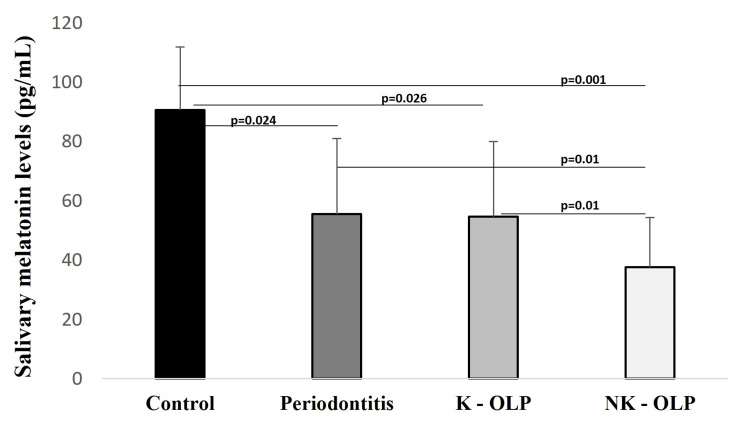
Salivary melatonin levels among the study groups. The statistical difference was observed between Control and periodontitis, keratotic oral lichen planus (K-OLP), non-keratotic oral lichen planus (NK-OLP); between periodontitis and NK-OLP; and between K-OLP and NK-OLP).

Melatonin was found in all the study samples. The salivary melatonin concentration appeared to decrease in saliva notably from controls to periodontitis to OLP patients (Figure 3). The melatonin levels in controls (mean ± SD; 90.582 ± 21.355) showed significant differences with periodontitis (p=0.024); K-OLP (p=0.026) and NK-OLP (p=0.001). Periodontitis groups showed salivary melatonin levels (mean ± SD; 55.463 ± 25.480) more than K-OLP (mean ± SD; 54.719 ± 25.227) and NK-OLP (mean ± SD; 37.658 ± 16.760) but the difference was not significant with K-OLP (p=0.30). However, the periodontitis group showed a statistically significant difference with NK-OLP (p=0.01). Comparison of salivary melatonin levels between K-OLP and NK-OLP presented significant differences (p=0.01). The least significant differences in the salivary melatonin between the study groups were observed between groups. The result showed substantial differences between controls and periodontitis and OLP groups. A notable difference observed between periodontitis and NK-OLP groups; K-OLP and NK-OLP groups. No significance was observed between periodontitis and K-OLP groups. Further, a comparison of the clinical parameters with the salivary melatonin levels revealed non-statistical differences.

## Discussion

The present study was conducted to determine the salivary melatonin levels among controls, periodontitis, and OLP patients. The study showed a decrease in the salivary flow rate and salivary melatonin level among study groups. The highest salivary melatonin level was observed in the healthy group while the keratotic and non-keratotic OLP patient group showed a consistent decrease in melatonin levels. The result provided a basis for understanding that decreased salivary flow and salivary melatonin can alter oral epithelial homeostasis and can accelerate oral diseases like OLP.

As known, periodontal disease is a common inflammatory condition, that affects the periodontal tissues, and the bacterial toxic products impact the host immune system [14]. It is suggested that the etiologic factors are responsible for disturbing the physiologic lipid peroxidation, which results in low-level antioxidant protection of periodontal tissues. The oxidative stress triggers break out in soft tissues of the periodontium via collagen destruction and bone tissue resorption [17]. Melatonin is known to exert a protective role through its antioxidant and anti-inflammatory action, where it inhibits the nitric oxide (NO) and interleukin 6 (IL-6) production induced by bacterial lipopolysaccharide (LPS) [18]. Clinical studies on chronic periodontitis have shown higher levels of oxidative stress suggesting an interplay between proinflammatory cytokines, inflammatory mediators, and reactive oxygen species. Hence, causing tissue damage and alveolar bone destruction in chronic periodontitis [19-21]. The lower levels of salivary melatonin observed in our periodontitis groups are suggestive of more melatonin consumption in competing the oxidative damage. Also, the pocket depth and CAL were significantly higher compared to other groups due to the presence of local factors (dental deposits, plaques, microorganisms) that affected the periodontium and caused further tissue damage. The salivary flow rate was also lower compared to healthy controls indicating a change of oral microflora as the disease progressed.

**Figure 4:**
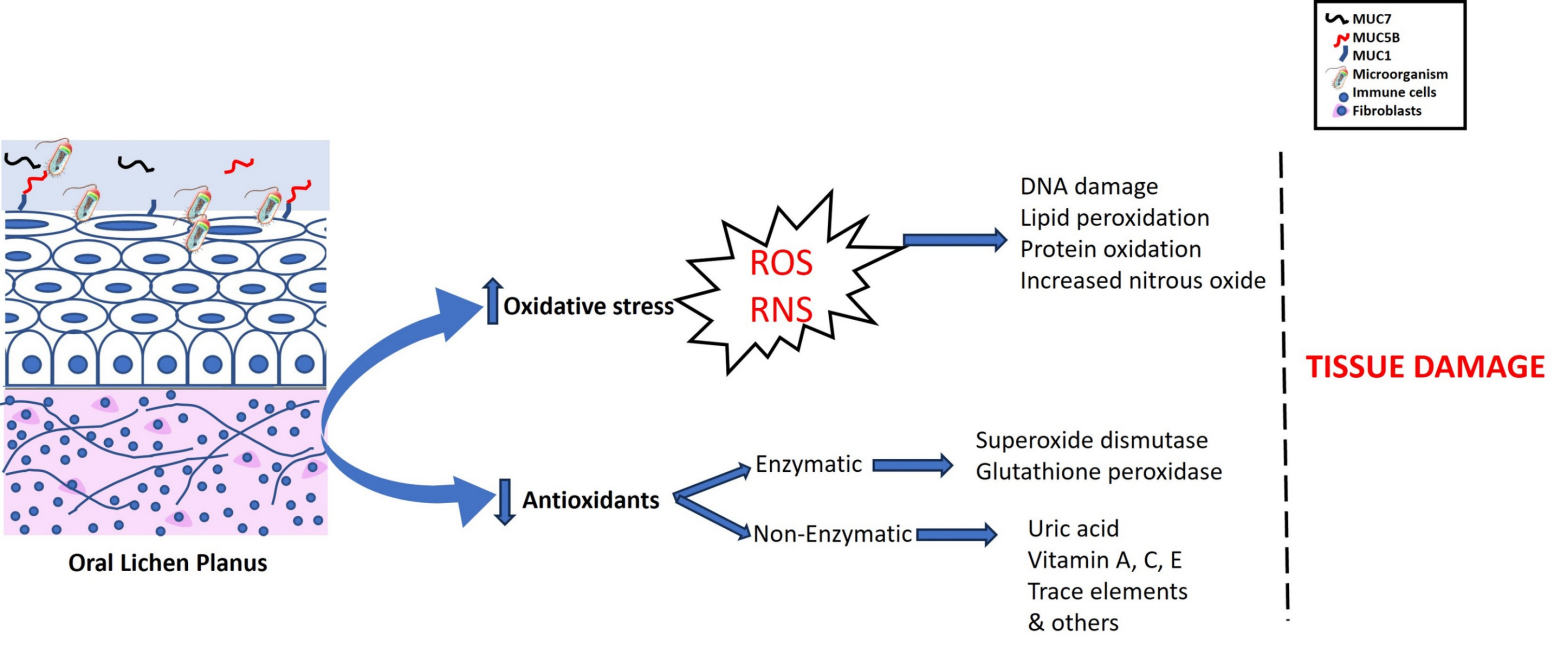
The imbalance in oxidant-antioxidant levels in oral lichen planus (OLP) is presented. The inflammatory cell infiltrates in OLP raise reactive oxygen species (ROS) and reactive nitrogen species (RNS) causing tissue damage via multiple mechanisms. The decrease in enzymatic and non-enzymatic antioxidants reflects a weaker defense against oxidative stress caused by OLP. Image credit: Bina Kashyap

OLP presents with chronic inflammation that creates oxidative stress on the oral tissues. The role of oxidative stress in the etiology of lichen planus has been studied earlier [22]. The lymphocytic inflammatory cell infiltrate in OLP is a potential source of ROS and therefore, oxidant-antioxidant imbalance is considered an effective mechanism in the OLP pathogenesis [23]. The oxidative stress can develop endogenous (via periodontitis) and exogenous (habits and environmental) that damage the tissues via multiple mechanisms, including DNA damage, lipid peroxidation (LPO) damage, and protein oxidation [24]. The increase in oxidative stress and the decrease in the antioxidant levels in OLP reflect the weak defense against oral tissues (Figure 4). The levels of oxidant and antioxidant markers were associated with OLP lesion severity [25]. However, the imbalance between oxidants and antioxidants is also observed in other oral diseases, including recurrent aphthous stomatitis (RAS) and periodontitis [26,27].

Emerging evidence has shown that elevated oxidative stress coupled with a lack of cellular repair processes is associated with mutation-induced carcinogenesis [25,28]. Melatonin assessment in our OLP patients has shown a significant decrease in keratotic and non-keratotic OLP compared to healthy controls. The non-keratotic OLP presented with lower salivary melatonin and decreased salivary flow rate compared to keratotic OLP. Our previous study confirmed a decrease in salivary flow rate and decreased salivary MUC5B in OLP patients [29]. Hence, referring to our previous and current findings it can be stated that the oral defense system is compromised in OLP patients. The antioxidant action of melatonin is decreased in the saliva, although its significant presence in the oral mucosal epithelial tissues is shown in another study. The melatonin biosynthesis in the oral epithelial tissue of OLP is attributed to the underlying lymphocytic infiltrate and other immune cells in the connective tissue. The lymphocyte-produced melatonin can modulate the IL-2/IL-2 receptor system and macrophage-produced melatonin can induce NF-kB ligand [30,31]. Based on these findings, it is possible that the continuous action of immune cells in response to stimuli and more production of ROS could alter the melatonin action in OLP. This further affects epithelial homeostasis and increases the chance of oral infection and oral diseases. The decreased salivary melatonin (due to decreased synthesis from the salivary gland) and a decreased salivary flow rate can enhance microbial colonization in the oral mucosal epithelium of OLP. Such change could result in varied oral symptoms and can contribute to various OLP manifestations. More recently, saliva microbiome dysbiosis studies in patients affected by OLP showed diverse microbiomes in different clinical forms of the OLP disease [32]. The present study has also observed no salivary melatonin difference between periodontitis and keratotic OLP patients, although clinical parameters showed some differences. As periodontitis etiology is influenced by oxidative stress which could correlate to keratotic OLP and in both cases, salivary melatonin fails to scavenge free radicals.

There is currently not much information available on oral melatonin and its importance in oral mucosal homeostasis and oral diseases. The results of the present study provide information that encourages upcoming research projects to investigate the biological functions of melatonin in response to chronic inflammation in oral mucosal tissues. Our study had some limitations: First, the study sample size was small. Second, the study did not investigate the details of habit (tobacco, alcohol, etc) history which could be an additional factor for disease progression and change of salivary melatonin levels in our study groups.

## Conclusions

To summarize, the present study confirms the salivary melatonin assessment as an easy and non-invasive method that reflects the real situation of the local oral environment. The uniqueness of the present study is that it provides information on salivary melatonin levels that can be used as a potential biochemical marker for the detection and progression of oral diseases. Additionally, more clinical and animal studies are necessary to determine the effectiveness of melatonin in the form of gels, toothpaste, and oral rinses in the clinical setting, aiming to prevent oral symptoms and early-stage oral disease progression.
